# DExH-box helicase 9 modulates hippocampal synapses and regulates neuropathic pain

**DOI:** 10.1016/j.isci.2024.109016

**Published:** 2024-01-25

**Authors:** Li Yang, Qiaoqiao Liu, Yaxuan Zhao, Ninghua Lin, Yue Huang, Qihui Wang, Kehui Yang, Runa Wei, Xiaotong Li, Ming Zhang, Lingyun Hao, Hongjun Wang, Zhiqiang Pan

**Affiliations:** 1 Jiangsu Province Key Laboratory of Anesthesiology, Xuzhou Medical University, Xuzhou, Jiangsu 221004, China; 2 Jiangsu Province Key Laboratory of Anesthesia and Analgesia Application Technology, Xuzhou Medical University, Xuzhou, Jiangsu 221004, China; 3 NMPA Key Laboratory for Research and Evaluation of Narcotic and Psychotropic Drugs, Xuzhou, Jiangsu 221004, China; 4 Department of Anesthesiology, Nanjing Drum Tower Hospital, The Affiliated Hospital of Nanjing University Medical School, Zhongshan Road 321, Nanjing 210008, China

**Keywords:** Natural sciences, Biological sciences, Physiology, Pathophysiology, Neuroscience, Systems neuroscience, Sensory neuroscience

## Abstract

Experimental studies have shown that neuropathic pain impairs hippocampal synaptic plasticity. Here, we sought to determine the underlying mechanisms responsible for synaptic changes in neuropathic painful mouse hippocampal neurons. Beyond demonstrating proof-of-concept for the location of DExH-box helicase 9 (DHX9) in the nucleus, we found that it did exist in the cytoplasm and DHX9 depletion resulted in structural and functional changes at synapses in the hippocampus. A decrease of DHX9 was observed in the hippocampus after peripheral nerve injury; overexpression of DHX9 in the hippocampus significantly alleviated the nociceptive responses and improved anxiety behaviors. Mimicking DHX9 decrease evoked spontaneous pain behavioral symptoms and anxiety emotion in naïve mice. Mechanistically, we found that DHX9 bound to *dendrin* (*Ddn*) mRNA, which may have altered the level of synaptic- and dendritic-associated proteins. The data suggest that DHX9 contributes to synapses in hippocampal neurons and may modulate neuropathic pain and its comorbidity aversive emotion.

## Highlights

• Proof-of-concept for the location of DHX9 in the nucleus, it did exist in the cytoplasm
• DHX9 depletion resulted in hippocampal synapse structural and functional changes
• DHX9 contributed to neuropathic pain and its comorbidity emotion
• DHX9 regulated DDN protein via binding to *Ddn* mRNA

## Graphical abstract

**Figure:**
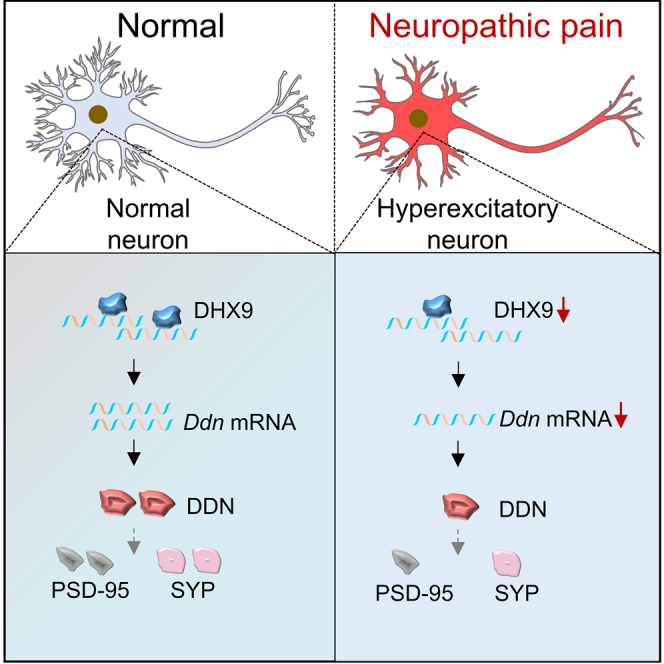

## Introduction

Neuropathic pain is a chronic and refractory disease that affects approximately 7%–10% of the adult population^1^^,^^2^ and severely compromises patients’ quality of life.^3^ To date, there are few safe and effective therapeutic agents because of the complex mechanisms underlying neuropathic pain and because its occurrence and development result from a combination of sensations and emotions related to the damage.^4^ Thus, advancing pain relief or elimination remains a goal that could benefit a huge number of patients.

Multiple brain regions participate in nociceptive processing.^5^ Of these, the hippocampus is a critical region for emotional processing and pain modulation.^6^^,^^7^^,^^8^ Patients with neuropathic pain have disordered functional connectivity networks that include the hippocampus, and the structure and function of hippocampal neurons are altered in a rat model of neuropathic pain.^9^^,^^10^ Furthermore, peripheral nerve injury-induced neuropathic pain can be inhibited by manipulation of the hippocampus in rodents.^11^^,^^12^ Chronic pain arising from nerve injury, including neuropathic pain, impairs long-term potentiation (LTP) at CA3–CA1 synapses and contributes to the loss of glutamatergic synapses in the hippocampus.^13^^,^^14^ These findings suggest that plastic changes at hippocampal synapses may induce neuropathic pain. Despite their ubiquity, the molecular mechanisms controlling synaptic changes remain elusive.

Superfamily 2 (SF2) helicases are critical molecular motors that participate in virtually all processes involved in DNA and RNA metabolism.^15^^,^^16^ Altered expression of one or more SF2 helicase contributes to psychiatric and neurological diseases such as depression, Alzheimer’s disease, Parkinson’s disease, multiple sclerosis, and amyotrophic lateral sclerosis.^17^^,^^18^^,^^19^^,^^20^^,^^21^ A recent study demonstrated that eIF4AIII, an SF2 helicase, modulates synaptic strength and neuronal protein expression by mediating cytoplasmic mRNA metabolism via a host of *trans*-acting factors that, together with the mRNA, form a messenger ribonucleoprotein particle (mRNP)^22^ that may participate in the pathophysiology of psychiatric and neurological diseases, including neuropathic pain. In another example, helicase–primase inhibition by amenamevir suppressed the development of acute herpetic pain and postherpetic neuralgia.^23^ DExH-box helicase 9 (DHX9) is an SF2 helicase with a robust role in DNA replication, transcription, translation, RNA processing and transport, microRNA processing, and the maintenance of genomic stability.^24^ In addition, the *Dhx9* gene was listed as a potential pathogenic gene for major depressive disorder.^19^ However, whether DHX9 modulates hippocampal synapses in an eIF4AIII-like manner, or even whether it participates in the pathology of neuropathic pain, remains unknown.

In the present study, we found that DHX9 has an eIF4AIII-like distribution pattern in hippocampal neurons. Decreased hippocampal DHX9 caused structural alterations and synaptic dysfunction, with the downregulation of the genes encoding dendritic-associated proteins. Interestingly, we observed a significant decrease in DHX9 in the hippocampus of mice with peripheral nerve injury and neuropathic pain. Mimicking this decrease in uninjured mice evoked neuropathic pain-like symptoms and anxiety emotion. Overexpression of DHX9 in mice with neuropathic pain notably attenuated their pain behaviors and comorbidity anxiety emotion. Our findings enrich the current literature on the functional link between DHX9 and synapses in the context of pain and provide evidence that DHX9 may be a novel therapeutic target for peripheral nerve injury-induced neuropathic pain.

## Results

### DExH-box helicase 9 is colocalized with both somatic and dendritic proteins in hippocampal neurons

**Figure 1:**
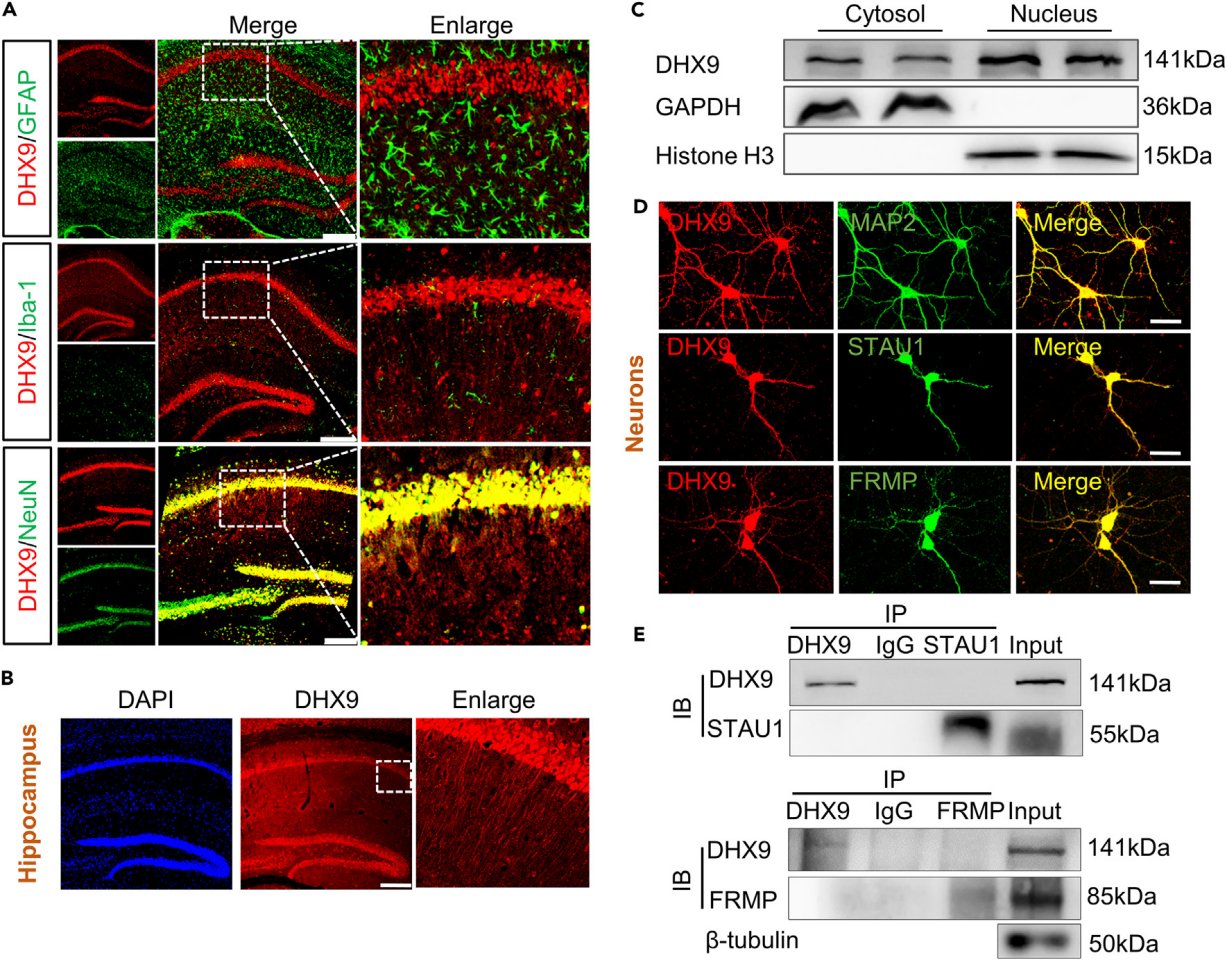
DHX9 exhibits somatodendritic localization in neurons and colocalizes with FRMP and STAU1 (A) Costaining of GFAP, Iba-1, or NeuN (green) with DHX9 (red) in the hippocampus of mice. Scale bar, 200 μm. (B) Coronal hippocampal section stained for DNA (blue) and DHX9 (red). The CA1 field (white box) is shown enlarged in the right panel, showing endogenous DHX9. Scale bar, 200 μm. (C) DHX9 expression in the naive mouse hippocampus cytoplasm and nucleus. n = 5 samples for each group. (D) Colocalization of endogenous DHX9 (red) with endogenous MAP2, FRMP, or STAU1 (green) in cultured hippocampal neurons. Scale bar, 50 μm. (E) Biochemical association of endogenous DHX9 (red) with endogenous FRMP or STAU1 in hippocampus.

SF2 helicases are critical for DNA and RNA metabolism^16^ and participate in myelination, neurogenesis, and synaptogenesis.^25^^,^^26^^,^^27^ Studies have shown that eIF4AIII, an SF2 helicase, is involved in the modulation of synaptic strength.^22^ To investigate whether a different SF2 helicase, DHX9, plays an eIF4AIII-like role in the regulation of synaptic plasticity, we assessed the distribution of DHX9 in coronal hippocampal sections. We found that DHX9 was co-localized with the neuronal marker NeuN in the hippocampus, but not with GFAP (a specific marker of astrocytes) or Iba-1 (a marker for microglia) (Figure 1A). Further verification of DHX9 distribution by immunofluorescence staining as well as the extraction of nuclear and cytoplasm showed that DHX9 was present not only in the nuclei but also in the cytoplasm of hippocampal neurons (Figures 1B and 1C). Next, we carried out immunofluorescence of DHX9 and dendritic proteins in primary hippocampal neurons. As expected, in primary cultured neurons, DHX9 displayed both somatic and dendritic staining and was colocalized with microtubule-associated protein 2 (MAP2), a motor protein expressed in both soma and dendrites, and two well-known dendritic-associated proteins, fragile X messenger ribonucleoprotein (FMRP), a delivery protein, and staufen double-stranded RNA binding protein 1 (STAU1), which represses dendritic mRNA (Figure 1D). To explore whether DHX9 associates with mRNPs (FRMP/STAU1), we performed Co-IP experiment. Unexpectedly, there is no biochemical association between DHX9 and FRMP, or DHX9 and STAU1 (Figure 1E).

### Downregulation of DExH-box helicase 9 induces alterations in dendritic properties and neuronal function properties

**Figure 2:**
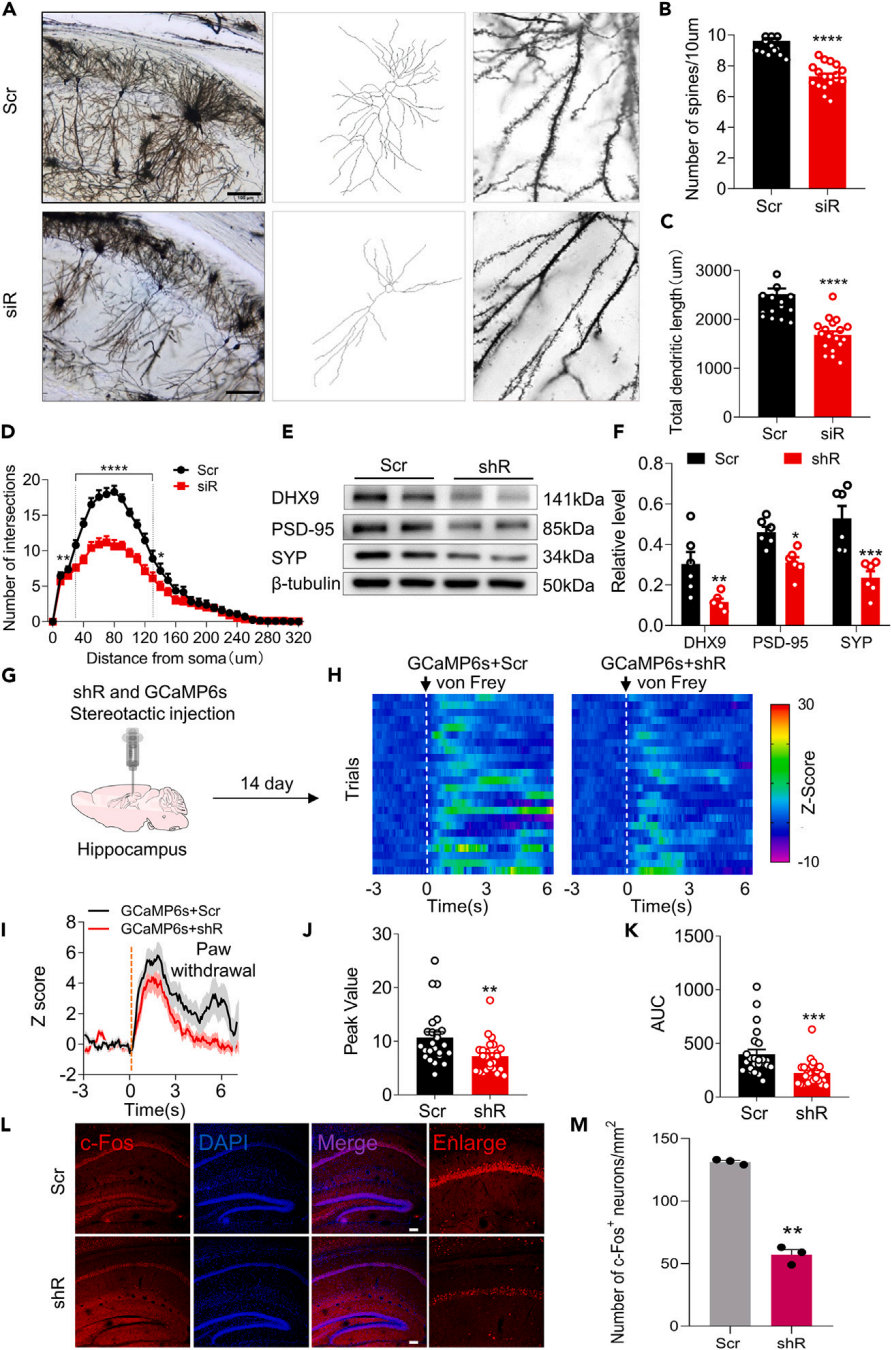
DHX9 knock-down alters synaptic properties (A–D) Representative images of z stack projections with Golgi–Cox staining showing neurons in the hippocampus at day 5 after the microinjection of *Dhx9*-siRNA (siR) or the scrambled control (Scr) (A), followed by analysis of the number of dendritic spines (B), dendritic length (C), and the number of intersections (Sholl analysis, D). Eighteen neurons from 3 animals/group. ∗∗∗∗p < 0.0001 versus the scrambled control group, Student’s *t* test. Scale bar, 100 μm. (E and F) Western blot analysis of DHX9, PSD-95, and SYP expression in *Dhx9*-shRNA (shR) treated hippocampus. Two representative immunoblots are presented from 6 mice/group. ∗p < 0.05, ∗∗p < 0.01, ∗∗∗p < 0.001 versus the Scr control group, Student’s *t* test. (G) Schematic of the calcium-imaging method. (H and I) Individual (H) and averaged (I) normalized GCaMP6s signals aligned to onset of von Frey filament stimulation of the ipsilateral hind paw. (J and K) Quantification of changes in the GCaMP6s signal: peak value (J) and area under the curve (AUC) (K). Twenty-four trials from 3 animals/group. ∗∗p < 0.01, ∗∗∗p < 0.001 versus the Scr group, Student’s *t* test. (L) Effect of DHX9 downregulation on neuronal activity. Representative images of neuron immunostaining for c-Fos in the naive mouse hippocampus. Scale bar = 100 μm. (M) Quantification of c-Fos-positive cells per mm^2^ in the hippocampus. Signals from 3 animals/group. ∗∗p < 0.01 versus the Scr group, Student’s *t* test.

To further determine the role of DHX9 in hippocampal synaptic plasticity, we evaluated the morphology of dendritic spines through Golgi–Cox staining after the hippocampal microinjection of *Dhx9*-specific RNA (siRNA) or its scrambled siRNA control. Downregulation of DHX9 resulted in a significant decrease in spine numbers, dendritic length, and the number of neuronal intersections in the hippocampus (Figures 2A–2D). Moreover, the hippocampal levels of two critical proteins, postsynaptic density protein-95 (PSD-95) and synaptophysin (SYP) were lower after the microinjection of lentivirus-expressed *Dhx9*-short hairpin RNA (shRNA) than after the injection of the scrambled control (Figures 2E and 2F).

Next, we examined the effect of DHX9 on neuronal activity. We used calcium imaging via AAV-CaMKIIα-GCaMP6s virus to gauge the population response of hippocampal neurons after the microinjection of *Dhx9*-shRNA or its scramble control (Figure 2G). As shown in Figures 2H–2K, the GCaMP6s signal in response to nociceptive mechanical stimulation (von Frey filament) was lower in the *Dhx9*-shRNA-treated group than in the corresponding scrambled control. The findings that the mark reduction of c-Fos^+^ neurons in *Dhx9-shRNA*-treated hippocampus provided strong evidence that the loss of function of DHX9 inhibited neuronal activity (Figures 2L and 2M). Together, these morphological and functional data indicate that the downregulation of hippocampal DHX9 alters dendritic complexity, spine density, and neuronal function.

### Decreased DExH-box helicase 9 is responsible for the diminished expression of dendritic-associated proteins in the hippocampus

**Figure 3:**
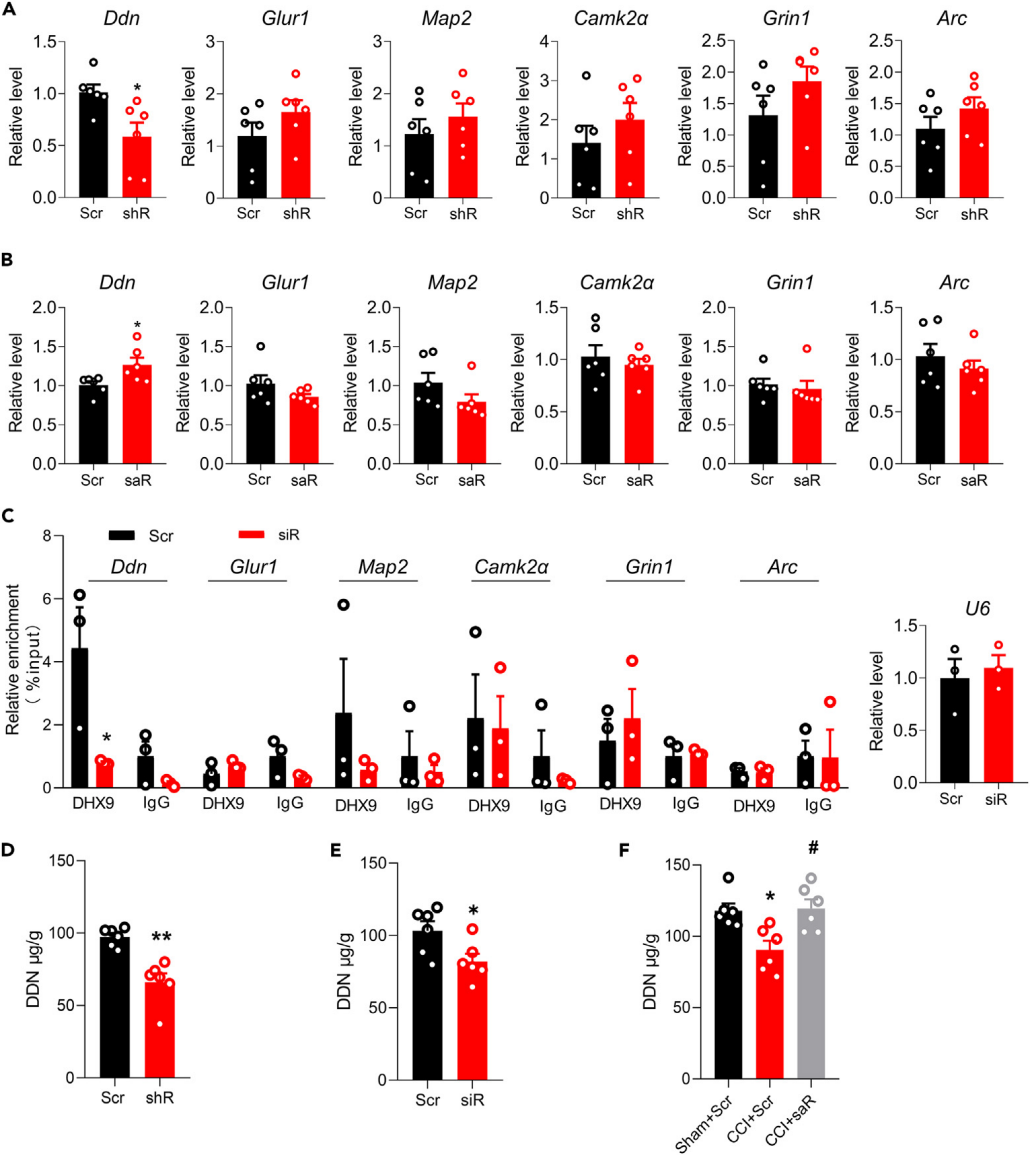
DHX9 is associated with *Ddn* (A) qPCR assay of dendritic mRNAs expression of different mRNAs in the hippocampus 2 weeks after shR injection, relative to the Scr control. n = 6 samples for each group. ∗p < 0.05 versus the Scr group, Student’s *t* test. (B) Relative expression of dendritic mRNAs in the *Dhx9*-saRNA (saR)-microinjected hippocampus, as determined by qPCR. n = 6 samples for each group. (C) Relative enrichment of endogenous dendritic mRNAs immunoprecipitated with DHX9 antibody in the siRNA-microinjected hippocampus as measured by RIP-qPCR. IgG as negative control, *U6* RNAs was used as target reference control. n = 3 samples for each group. ∗p < 0.05 versus the Scr group, Student’s *t* test. (D and E) The level of hippocampal DDN in naive mice microinjected with shRNA (D) or siRNA (E) as measured by ELISA. n = 6 samples for each group. ∗p < 0.05, ∗∗p < 0.01 versus the corresponding control group, Student’s *t* test. (F) The effect of DHX9 on hippocampal DDN in CCI mice treated with saRNA as measured by ELISA. n = 6 samples for each group. F (2, 15) = 7.578. ∗p < 0.05 versus the Sham+Scr group; ^#^p < 0.05 versus the CCI+Scr group, one-way ANOVA followed by Holm–Sidak post hoc multiple-comparisons test.

To identify the molecular mechanisms underlying the DHX9-involved synaptic changes, we measured the expression levels of six dendritic mRNAs by qPCR, as in a previous study on eIF4AIII,^22^ another SF2 helicase that has similar subcellular localization to DHX9. As shown in Figure 3A, the expression of *Ddn*, which is enriched in dendritic spines, was repressed after treatment with *Dhx9*-shRNA, but this was not the case for *Glur1*, *Map2*, *Camk2α*, *Grin*, or *Arc*. When DHX9 was overexpressed by treatment with a *Dhx9*-specific small active RNA (saRNA), an increase in *Ddn* expression was observed relative to the scrambled control (Figure 3B). Furthermore, we performed cytosolic RIP-PCR to explore whether DHX9 acts directly on *Ddn* mRNA. After *Dhx9*-siRNA microinjection, we observed that, among the six mRNAs, only the *Ddn* signal was decreased (Figure 3C). Next, we performed ELISA to examine the level of DDN protein. As shown in Figures 3D and 3E, downregulated DHX9 with shRNA or siRNA significantly decreased the level of DDN, and upregulated DHX9 with saRNA significantly rescued CCI-induced the decrease of DDN in the hippocampus (Figure 3F). These data suggest that DHX9 may effect synaptic changes by binding to *Ddn* mRNA.

### DExH-box helicase 9 is decreased in the contralateral hippocampus after peripheral nerve injury

**Figure 4:**
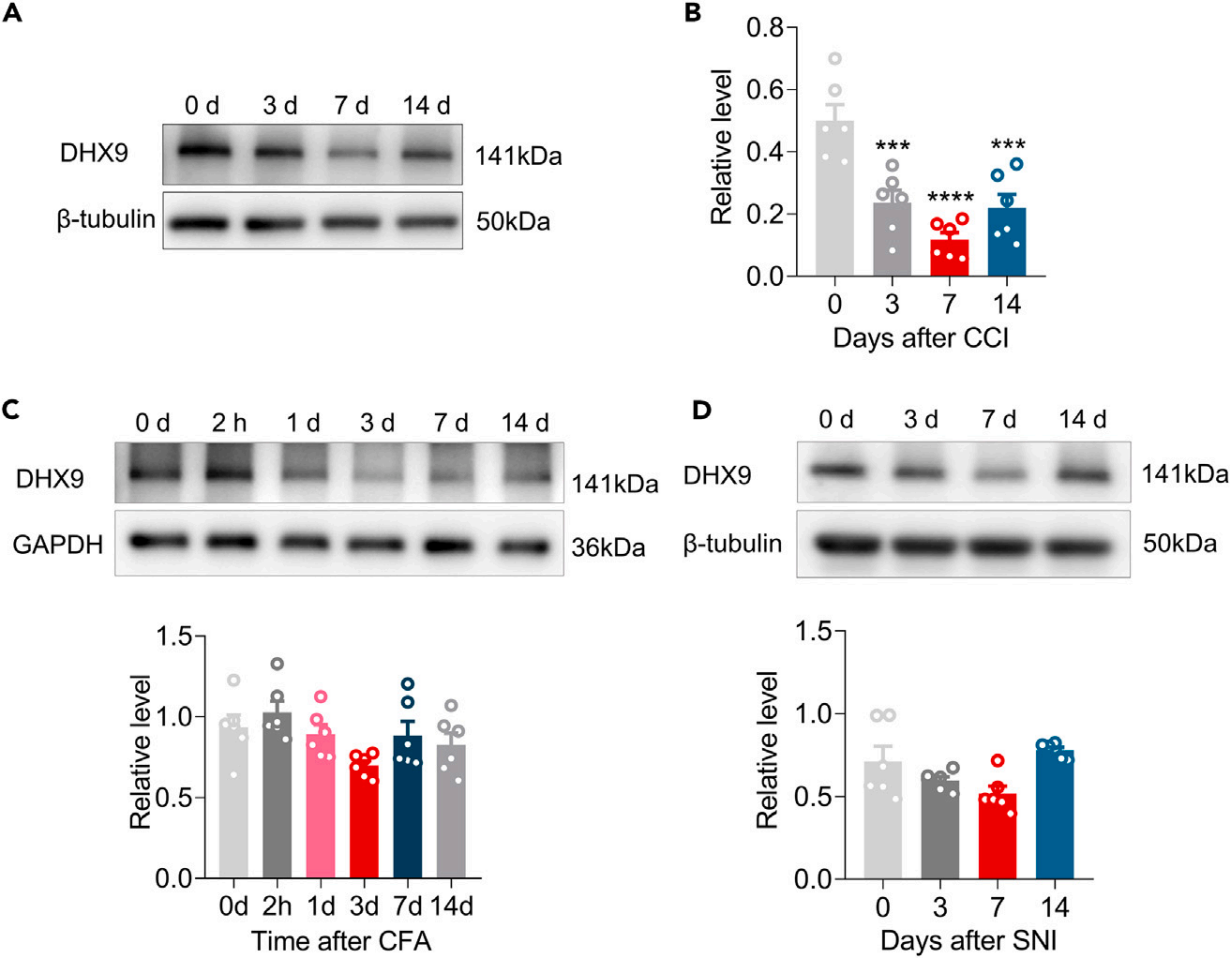
Downregulation of DHX9 in the hippocampus of mice with peripheral nerve injury-induced pain (A and B) Western blot assay of DHX9 expression level in the hippocampus of CCI mice on days 0, 3, 7, and 14 after surgery. n = 6 samples for each group. F (3, 20) = 15.66, ∗∗∗p < 0.001, ∗∗∗∗p < 0.0001 versus the 0 days group, one-way ANOVA followed by Holm–Sidak post hoc multiple-comparisons test. (C and D) Western blot assay of DHX9 expression in the hippocampus of mice with CFA-induced inflammatory pain (C) or SNI-evoked neuropathic pain (D). n = 6 samples for each group, one-way ANOVA followed by Holm–Sidak post hoc multiple-comparisons test.

Central sensitization in chronic pain is associated with synaptic plasticity so we next sought to investigate the potential role of DHX9 in neuropathic pain. We examined the expression of DHX9 in the contralateral hippocampus after chronic constriction injury (CCI) of the sciatic nerve in mice. DHX9 expression was significantly reduced in the contralateral hippocampus on day 7 after unilateral CCI (Figures 4A and 4B). With CFA-induced inflammatory pain, a trend toward lower DHX9 expression was observed in the contralateral hippocampus on day 3, but the reduction did not reach significance (Figure 4C). Similar results were observed in the contralateral hippocampus on day 3 after unilateral SNI (Figure 4D). Collectively, our findings indicate that the downregulation of DHX9 might be universal in pain, especially in CCI-induced neuropathic pain.

### Rescuing the downregulation of hippocampal DExH-box helicase 9 alleviates nociceptive responses

**Figure 5:**
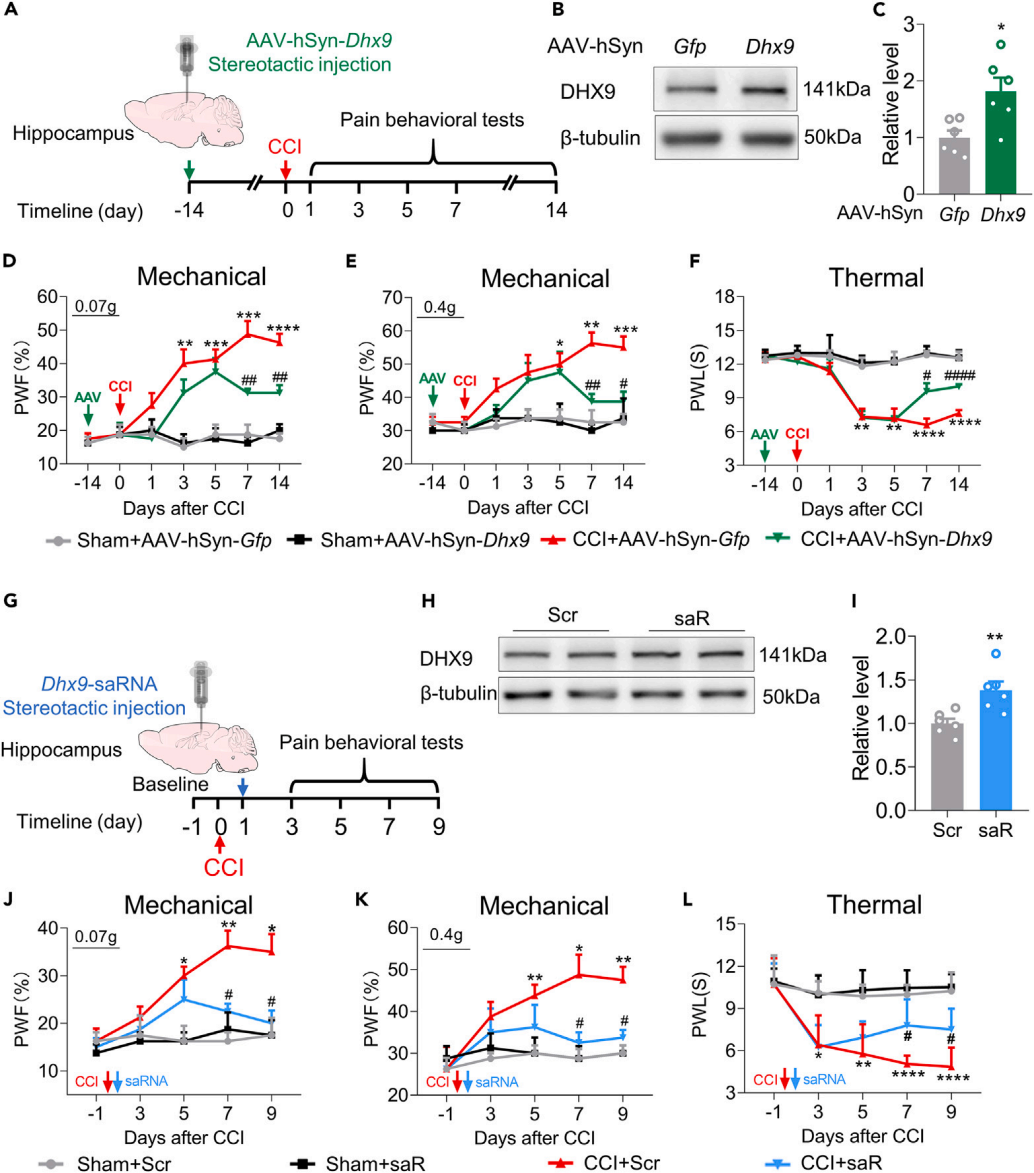
Overexpression of DHX9 ameliorates CCI-induced mechanical and thermal allodynia (A) Schematic and timeline of the AAV overexpression experiment. (B and C) Western blot analysis of DHX9 expression in AAV-hSyn-*Dhx9* injected hippocampus. n = 6 samples for each group. ∗p < 0.05 versus the *Gfp* group, Student’s *t* test. (D–F) DHX9 overexpression reversed the hypersensitivity to mechanical and thermal stimuli at different time points after CCI, as measured by the von Frey filament test (D and E) and the heat test (F). n = 8 samples for each group. Time factor: F (5.016, 140.5) = 17.84, group factor: F (3, 28) = 23.97 for (D); Time factor: F (4.829, 135.2) = 7.468, group factor: F (3, 28) = 14.30 for (E); Time factor: F (4.064, 113.8) = 20.03, group factor: F (3, 28) = 25.74 for (F). ∗p < 0.05, ∗∗p < 0.01, ∗∗∗p < 0.001, ∗∗∗∗p < 0.0001 versus the Sham+AAV-*Gfp* group; ^#^p < 0.05, ^##^p < 0.01, ^####^p < 0.0001 versus the CCI+AAV-*Gfp* group, two-way repeated-measures ANOVA followed by Holm–Sidak post hoc multiple-comparisons test. (G) Schematic and timeline for the experiment with the overexpression of DHX9 by saRNA. (H and I) Western blot analysis of DHX9 expression in saRNA injected hippocampus. n = 6 samples for each group. ∗p < 0.05 versus the Scr control group, Student’s *t* test. (J–L) DHX9 overexpression alleviated nociceptive responses at multiple time points after CCI, as measured by the von Frey filament test (J-K) and the heat test (L). n = 8 samples for each group. Time factor: F (2.927, 81.97) = 5.648, group factor: F (3, 28) = 17.69 for (J); Time factor: F (2.691, 75.36) = 6.152, group factor: F (3, 28) = 6.440 for (K); Time factor: F (2.163, 60.55) = 18.27, group factor: F (3, 28) = 24.64 for (L). ∗p < 0.05, ∗∗p < 0.01, ∗∗∗∗p < 0.0001 versus the Sham+Scr group; ^#^p < 0.05 versus the CCI+Scr group, two-way repeated-measures ANOVA followed by Holm–Sidak post hoc multiple-comparisons test.

To investigate the role of hippocampal DHX9 in neuropathic pain, we first pretreated the mouse hippocampus with AAV expressing full-length *Dhx9* (AAV-hSyn-*Dhx9*), as illustrated in Figure 5A. Two weeks later, the efficacy of AAV-hSyn-*Dhx9* was validated, as shown in Figures 5B and 5C. As expected, the expression of DHX9 was significantly increased in the AAV-hSyn-*Dhx9*-microinjected group compared with the AAV-*Gfp* group. CCI induced mechanical allodynia in the control group but this was notably ameliorated by prior DHX9 overexpression (Figures 5D and 5E). Similarly, as shown in Figure 5F, prior microinjection of AAV-hSyn-*Dhx9* significantly blocked the thermal hyperalgesia induced by CCI.

To investigate the potential therapeutic effect of DHX9 on pain, we employed a second DHX9 overexpression strategy utilizing *Dhx9*-saRNA. In this strategy, we microinjected *Dhx9*-saRNA or scrambled into the contralateral hippocampus on day 1 after CCI (Figure 5G). The efficiency of *Dhx9*-saRNA was first demonstrated by microinjecting it into naive mice: the group treated with *Dhx9*-saRNA had higher levels of DHX9 than the group treated with the scrambled control (Figures 5H and 5I). Hippocampal microinjection of *Dhx9*-saRNA in CCI mice blocked and reversed CCI-induced mechanical allodynia and heat hyperalgesia from day 7–9 post-CCI (Figures 5J–5L). This indicates that DHX9 has a therapeutic effect on pain-like behavior after peripheral nerve damage.

### Mimicking chronic constriction injury-induced downregulation of DExH-box helicase 9 in the hippocampus evokes neuropathic pain-like behaviors

**Figure 6:**
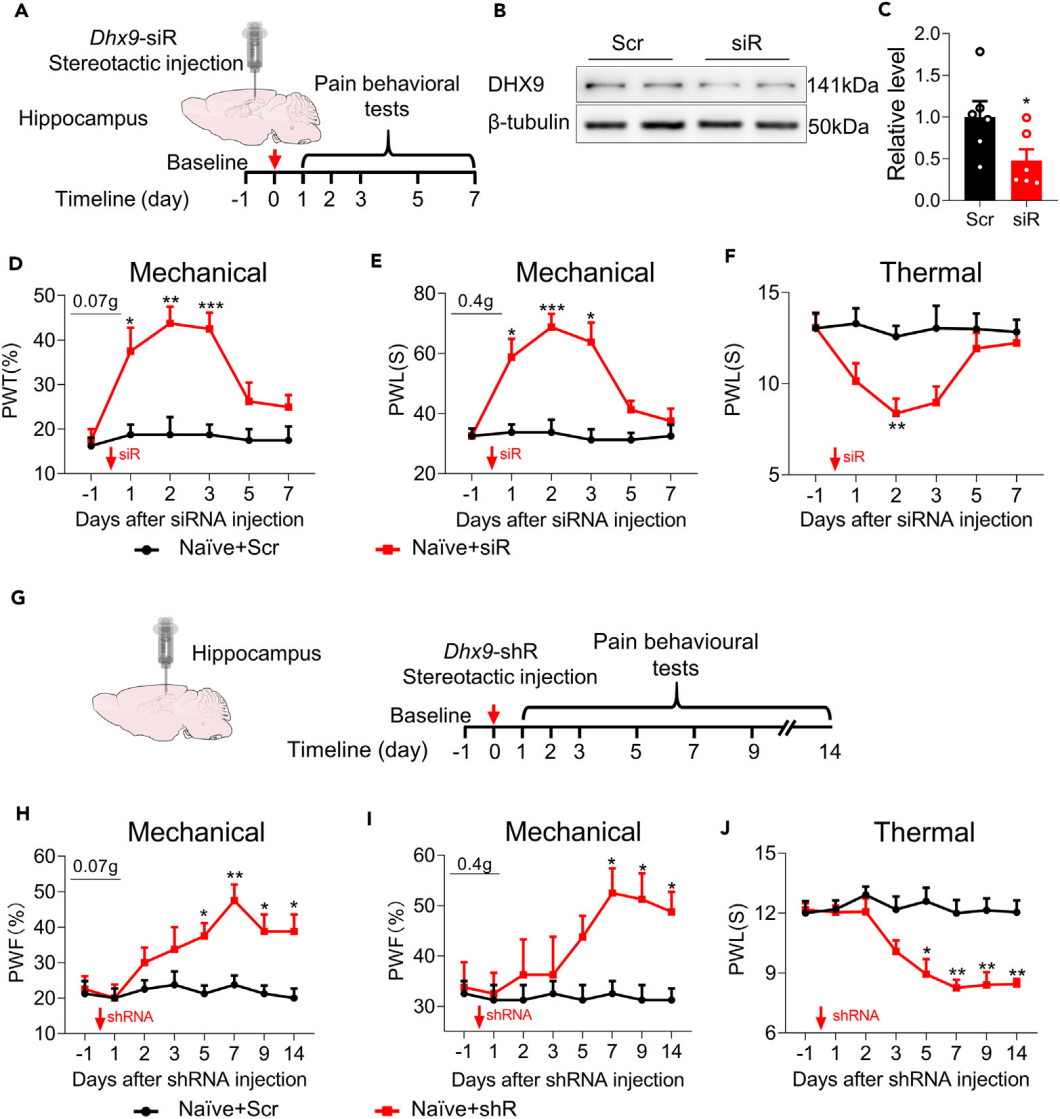
Mimicking CCI-induced downregulation of DHX9 in the hippocampus induces neuropathic pain-like behavior (A) Schematic and timeline of DHX9 knock-down in the hippocampus by siRNA and subsequent behavioral tests. (B and C) Western blot analysis of DHX9 expression in siRNA-injected hippocampus. n = 6 samples for each group. ∗p < 0.05 versus the Scr control group, Student’s *t* test. (D–F) Decreasing DHX9 in naive mice increased their sensitivity to mechanical and thermal stimuli, as measured by the von Frey filament test (D and E) and the heat test (F). n = 8 samples for each group. Time factor: F (3.760, 52.64) = 9.030, group factor: F (1, 14) = 21.41 for (D); Time factor: F (4.163, 58.28) = 10.17, group factor: F (1, 14) = 26.36 for (E); Time factor: F (4.354, 60.95) = 2.916, group factor: F (1, 14) = 9.457 for (F). ∗p < 0.05, ∗∗p < 0.01, ∗∗∗p < 0.001 versus the Naïve+Scr group, two-way repeated-measures ANOVA followed by Holm–Sidak post hoc multiple-comparisons test. (G) Schematic and timeline of DHX9 knock-down in the hippocampus by shRNA and subsequent behavioral tests. (H–J) DHX9 downregulation in naive mice evoked spontaneous pain, as measured by the von Frey filament test (H-I) and the heat test (J). n = 8 samples for each group. Time factor: F (3.462, 48.46) = 3.685, group factor: F (1, 14) = 25.69 for (H); Time factor: F (3.086, 43.20) = 2.237, group factor: F (1, 14) = 9.835 for (I); Time factor: F (3.735, 52.28) = 6.294, group factor: F (1, 14) = 45.23 for (J). ∗p < 0.05, ∗∗p < 0.01 versus the Naïve+Scr group, two-way repeated-measures ANOVA followed by Holm–Sidak post hoc multiple-comparisons test.

To further determine the effect of DHX9 on the induction and the maintenance of neuropathic pain, we microinjected *Dhx9*-siRNA into the unilateral hippocampus in naïve mice to mimic the CCI-induced hippocampal decrease in DHX9 (Figure 6A). *Dhx9*-siRNA administration significantly reduced the level of DHX9 in the hippocampus (Figures 6B and 6C). Microinjection of *Dhx9*-siRNA, but not its scrambled siRNA, induced mechanical allodynia from day 1–3 and thermal hyperalgesia at day 2 post-microinjection (Figures 6D–6F). To confirm these pain-like findings caused by the loss of function of DHX9, we microinjected *Dhx9*-shRNA into the unilateral hippocampus in naïve mice (Figure 6G). Consistent with the siRNA results, hippocampal microinjection of *Dhx9*-shRNA produced lasting mechanical allodynia from day 7 to day 14 post-injection, persisting for at least 7 days, and heat hyperalgesia from day 3 to day 14 post-injection, persisting for at least 11 days (Figures 6H–6J). Our results show that the downregulation of DHX9 in the hippocampus induces neuropathic pain-like behaviors in naïve mice, suggesting that hippocampal DHX9 may participate in the induction and development of neuropathic pain.

### DExH-box helicase 9 in the hippocampus contributes to anxiety-like behaviors

**Figure 7:**
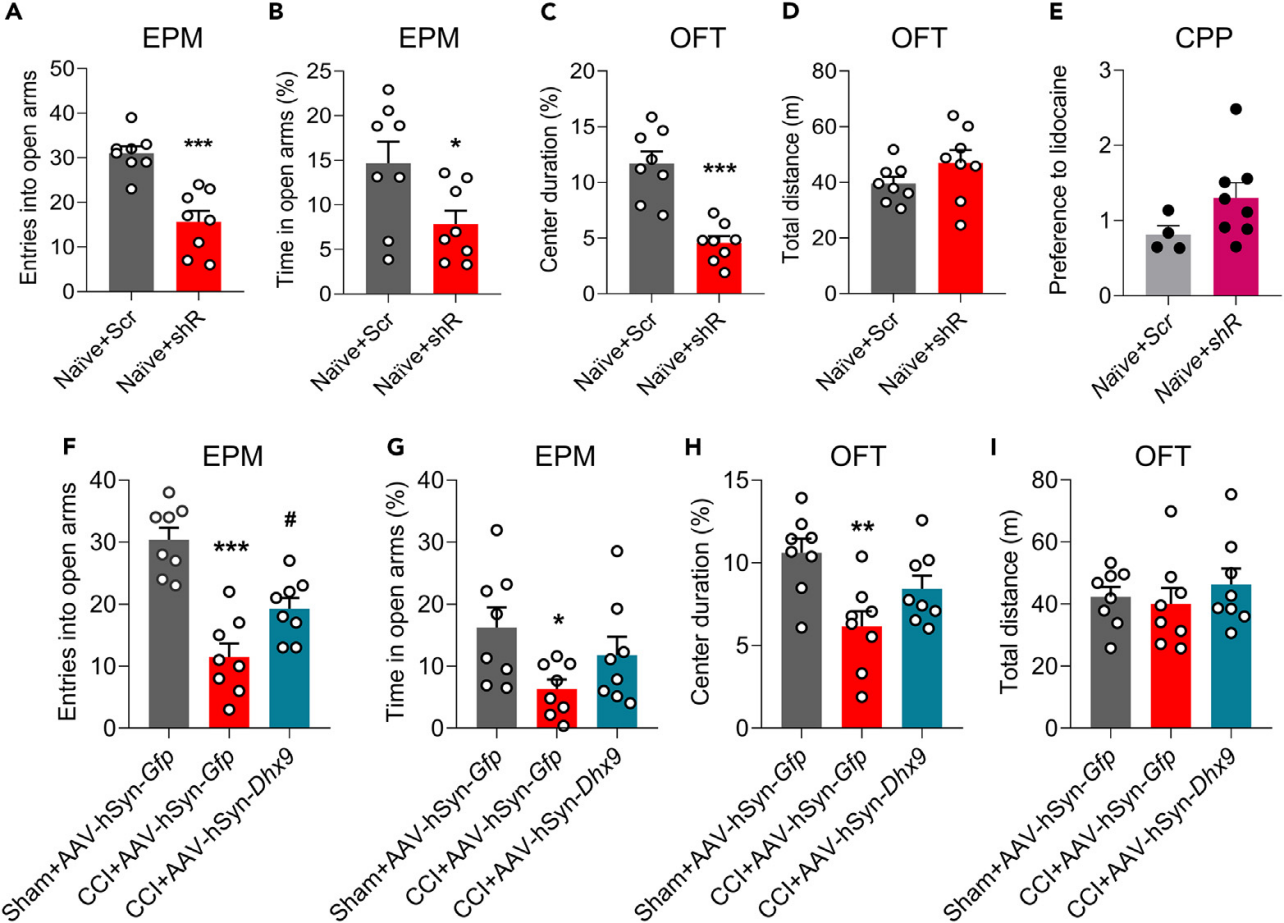
The contribution of DHX9 to anxiety-like behaviors (A–D) The effect of DHX9 downregulation on anxiety-like behaviors in the elevated plus maze (EPM) (B-C) and open field test (OFT) (D–E) in naive mice. n = 8 samples for each group. ∗p < 0.05, ∗∗∗p < 0.001 versus the Scr group, Student’s *t* test. (E) The effect of DHX9 downregulation on aversive memory of pain, as measured by the conditional position preference (CPP) test. n = 4 samples for Scr group, n = 8 samples for shRNA group. (F–I) The effect of DHX9 on CCI-induced anxiety-like behaviors in the EPM (F-G) and OFT (H-I). n = 8 samples for each group. F (2, 21) = 23.00 for (F); F (2, 21) = 3.440 for (G); F (2, 21) = 6.709 for (H). ∗p < 0.05, ∗∗p < 0.01, ∗∗∗p < 0.001 versus the Sham+AAV-*Gfp* group; ^#^p < 0.05 versus the CCI+AAV-*Gfp* group, one-way ANOVA followed by Holm–Sidak post hoc multiple-comparisons test.

Chronic pain is generally accompanied by aversive emotional states and cognitive deficits, which can be associated with impaired hippocampal synaptic plasticity.^28^ Thus, we firstly sought to examine if the dysfunction of hippocampal DHX9 could induce the genesis of the anxiety-like behaviors. As shown in Figures 7A and 7B, downregulation of hippocampus DHX9 deceased the entries into open arms and the time in open arms as evaluated by elevated plus maze (EPM) on day 7 after the injection of *Dhx9*-shRNA in naïve mice. Consistent with the findings, *Dhx9*-shRNA-treated mice stayed at a lower center duration compared with scrambled-treated mice in the open field test (OFT), despite of no difference in total distance (Figures 7C and 7D). Additionally, the cognitive behavioral data from the conditional position preference (CPP) test,^29^ showed that DHX9 knockdown established an inapparent memory deficit after the same treatment with *Dhx9*-shRNA in naïve mice (Figure 7E). It appears that hippocampus DHX9 may be only associated with aversive emotion. Moreover, we further assessed whether the upregulation of hippocampus DHX9 could alleviate the anxiety symptoms. Consistent with previous reports,^30^ nerve injury significantly anxiety-like behaviors as tested by the decreased entries into open arms and the time in open arms (Figures 7F–7H), and overexpression of DHX9 with AAV significantly revered the CCI-induced decrease of entries number into open arms (Figure 7F), and the bias in time in open arms (Figure 7G), compared with the *Gfp* group. Similar results were recorded with OFT, where animals injected with AAV-hSyn-*Dhx9* showed increased bias in center duration compared to AAV-hSyn-*Gfp* mice (Figure 7H); however, there were no differences in the total distance between the AAV-hSyn-*Dhx9* and AAV-hSyn-*Gfp* groups (Figure 7I). Collectively, the findings suggested that hippocampus DHX9 might be involved in pain-related anxiety emotion genesis.

## Discussion

SF2 helicases that have robust functions related to synaptic plasticity offer potential treatment strategies for neuropsychiatric diseases. In the present study, we identified a novel regulator of synaptic plasticity, DHX9, which is a member of the SF2 helicases. DHX9 displays an eIF4AIII-like distribution pattern in hippocampal neurons. Downregulation of DHX9 caused a decrease in morphological dendritic properties and a reduction in the evoked neural response, and directly reduced the expression of *Ddn* in the hippocampus. Examination of the mechanism underlying these observations revealed that DHX9 binds with *Ddn* mRNA to regulate its expression. We also found that DHX9 expression is reduced after the induction of neuropathic chronic pain and that DHX9 administration significantly alleviates pain-like behaviors. Furthermore, manipulations that lower the level of DHX9 evoke hyperalgesia in naïve mice. This study sheds light on the function of DHX9 and provides new avenues for the development of preventive strategies and effective treatment of neuropathic pain.

Experimental studies have found that pain and memory deficits induced by peripheral nerve injury are linked to impaired LTP at CA1–CA3 synapses in the hippocampus^13^ and reduced dendritic lengths and spine densities in hippocampal CA1 neurons.^31^ Multiple studies have also shown that peripheral nerve injury induces neurogenesis in the dentate gyrus (DG) region of the hippocampus and that injecting lidocaine into the DG produces analgesia.^12^^,^^32^ We found that neuropathic pain after peripheral nerve injury leads to a decrease in DHX9 expression in the contralateral hippocampus. Mimicking this decrease caused neuropathic pain-like symptoms and anxiety-like symptoms in naive mice and alterations in properties linked to synaptic plasticity in hippocampal neurons. Consistent with previous studies, our findings support the view that the hippocampus is a critical region in the regulation of neuropathic pain and is involved in the affective aspects of pain perception and related emotional processes.^33^ Thus, it may be difficult to determine the precise role of the identified mechanisms and morphological changes in neuropathic pain or other diseases.

To our knowledge, this is the first study to demonstrate the functional link between DHX9 and DDN in the context of synaptic changes. In the hippocampus, *Ddn* mRNA is located in the soma and dendrites, and DDN is enriched in the postsynaptic density. DDN modulates LTP and dendritic spine numbers in CA1 pyramidal neurons via binding with kibra^34^ and is important for regulating the structure and function of the postsynaptic cytoskeleton.^35^^,^^36^^,^^37^ Moreover, weakened hippocampal synaptic plasticity is correlated with a clear decrease in *Ddn*.^38^ These findings suggest that DDN plays an important role in synaptic plasticity. In our study, localization of DHX9 was shown to keep pace with *Ddn*, and downregulating DHX9 decreased the expression of *Ddn*, evoking attenuated synaptic responses. We speculate that the DHX9 regulation of *Ddn* may mediate synaptic changes that lead to pain sensitivity.

PSD-95 and SYP have a strong association with synaptic plasticity.^39^^,^^40^ Interestingly, we detected significant decreases in PSD-95 and SYP expression after the downregulation of DHX9. It is well-known that PSD-95 modulates the formation of the postsynaptic density and affects synaptic plasticity via direct or indirect interactions with glutamate receptors and/or intracellular proteins.^41^^,^^42^ In neuropathic pain, the role of PSD-95 has been constantly redefined. Inhibiting a combination of PSD-95 and neuronal nitric oxide synthase (nNOS) or disrupting the interaction between PSD-95 and the NMDA receptor subunit NR2B both effectively relieved pain.^43^^,^^44^ SYP is an important component of the secretory granule membrane in neuroendocrine cells and synaptic vesicles in neurons. Deleting SYP causes a reduction in short-term and long-term synaptic plasticity in the CA1 region of the hippocampus.^45^ Changes in SYP have also been associated with changes in synaptic plasticity in chronic pain.^46^ Here, we found a statistically significant reduction in PSD-95 and SYP expression with DHX9 knock-down. Although we verified the relationship between DHX9 and *Ddn* mRNA in hippocampal extracts, we cannot rule out the possibility that mechanisms unrelated to DDN may underlie the ability of DHX9 to regulate PSD-95 and SYP.

In summary, our study demonstrates that decreased levels of DHX9 induce alterations in synaptic- and dendritic-associated genes or proteins and contribute to synaptic changes in the hippocampus. Decreased DHX9 expression was observed in a mouse model of neuropathic pain, and reducing DHX9 in naïve mice directly evoked pain-like symptoms and its comorbidity emotion. These data reveal potential roles of DHX9 in modulating synaptic plasticity and in the pathophysiology of neurological disorders that are characterized by synaptic abnormalities, such as neuropathic pain.

### Limitation of the study

The current study only described the modulation of hippocampal DHX9 in neuropathic pain, leaving the changes in other brain regions largely unclear. Although our previous study has revealed that dorsal horn DHX9 regulated the production of ciRNA-Fmn1 by binding to DNA-tandem repeats and contributed to the genesis of neuropathic pain,^47^ the specific regulatory mechanism underlying the role of DHX9 in hippocampal synaptic plasticity in the context of neuropathic pain is elusive.

## STAR★Methods

### Key resources table

**Table:** 

REAGENT or RESOURCE	SOURCE	IDENTIFIER
**Antibodies**		

Rabbit polyclonal anti-DHX9	Proteintech	Cat#17721-1-AP; RRID:AB_2092506
Mouse monoclonal anti-β-tubulin	Proteintech	Cat#66240-1-Ig; RRID:AB_2881629
Rabbit polyclonal anti-PSD95	HUABIO	Cat#R1508-4; RRID:AB_3073317
Rabbit monoclonal anti-synaptophysin	Abcam	Cat#ab32127; RRID:AB_2286949
Rabbit polyclonal anti-Histone H3	Abcam	Cat#ab1791; RRID:AB_302613
Mouse monoclonal anti-NeuN	Abcam	Cat#ab104224; RRID:AB_10711040
Goat polyclonal anti-Iba1	Abcam	Cat#ab5076; RRID:AB_2224402
Mouse monoclonal anti-GFAP	Sigma-Aldrich	Cat#G3893; RRID:AB_477010
Mouse monoclonal anti-MAP2	Abcam	Cat#ab11267; RRID:AB_297885
Mouse monoclonal anti-FRMP	Santa Cruz	Cat#sc-101048; RRID:AB_1122951
Mouse monoclonal anti-STAU1	Santa Cruz	Cat#sc-390820; RRID:AB_3076745
Mouse monoclonal anti-*c*-Fos	Abcam	Cat#ab208942; RRID:AB_2747772

**Bacterial and virus strains**		

AAV-CaMKIIα-GCaMP6s	BrainVTA	PT-0110
AAV-hSyn-Dhx9	This paper	N/A
AAV-Dhx9 plasmid	Miao Ling Biotechnology	M15911

**Chemicals, peptides, and recombinant proteins**		

complete Freund’s adjuvant	Sigma-Aldrich	Cat#F5881

**Critical commercial assays**		

PD Rapid GolgiStain Kit	FD NeuroTechnologies	Cat#PK401
Nuclear and Cytoplasmic Extraction Kit	CoWin Biosciences	Cat# CW0199S
AAVpro® Purification kit	Takara	Cat#6666
Centricon Plus-70 filter unit	Millipore	Cat#UFC910096
Catch & Release v2.0 Reversible Immunoprecipitation System	Millipore	Cat# 17-500
Mouse Dendrin ELISA kit	Meimian	Cat# MM-47575M2

**Experimental models: Organisms/strains**		

Mouse: Male BALB/c	Animal Science Center of Xuzhou Medical University	
Mouse: pregnant female C57BL/6J (E12-E13 days)	Animal Science Center of Xuzhou Medical University	

**Oligonucleotides**		

siRNA sequence: Dhx9 Sense: GCACGUGAACAUGGAUCUATT Antisense: UAGAUCCAUGUUCACGUGCTT	Genepharma	N/A
saRNA sequence: Dhx9 Sense: GCTAGCGAAUCUUGGGAAATT Antisense: UUUCCCAAGAUUCGCUAGCTT	Genepharma	N/A
Sequence: Scramble Sense: UUCUCCGAACGUGUCACGUTT Antisense: ACGUGACACGUUCGGAGAATT	Genepharma	N/A
shRNA targeting sequence: Dhx9 Forward: CGCGTGCACGTGAACATGGATCTAAGAG ATAGATCCATGTTCACGTGCTTTTTGGAAAT Reverse: CGATTTCCAAAAAGCACGTGAA CATGGATCTATCTCTTAGATCCATGTTCACGTGCA	Genepharma	N/A
PCR Primer: Dendrin Forward:GACCCTGGGGACTAAGCGA Reverse:ACATCCCGGTAGATTCGAGGA	Sangon Biotech	N/A
PCR Primer: Arc Forward:AAGTGCCGAGCTGAGATGC Reverse:CGACCTGTGCAACCCTTTC	Sangon Biotech	N/A
PCR Primer: Map2 Forward:ATGACAGGCAAGTCGGTGAAG Reverse:CATCTCGGCCCTTTGGACTG	Sangon Biotech	N/A
PCR Primer: Camk2α Forward:GAATCTGCCGTCTCTTGAA Reverse:TCTCTTGCCACTATGTCTTC	Sangon Biotech	N/A
PCR Primer: Glur1 Forward:ATGTGGAAGCAAGGACTCCG Reverse:TAGAACACGCCTGCCACATT	Sangon Biotech	N/A
PCR Primer: Grin1 Forward:AGAGCCCGACCCTAAAAAGAA Reverse:CCCTCCTCCCTCTCAATAGC	Sangon Biotech	N/A
PCR Primer: U6 Forward:CTCGCTTCGGCAGCACATATACT Reverse:ACGCTTCACGAATTTGCGTGTC	Sangon Biotech	N/A

**Software and algorithms**		

QuantStudio^TM^ 7 Flex PCR system	ThermoFisher scientific	https://www.thermofisher.cn/order/catalog/product/4485701
GraphPad Prism8.0	GraphPad Software, Inc.	https://www.graphpad.com/features
ImageJ 2.0.0	NIH	https://imagej.nih.gov/ij/download.html
Fiji-ImageJ	NIH	https://imagej.net/Fiji/Downloads

**Other**		

von Frey filaments (0.07 g and 0.4 g)	Stoelting, Wood Dale, IL.	N/A
Model 336 Analgesia Meter	IITC Inc. Life Science Instruments, Woodland Hills	N/A
HiScript II Q RT SuperMix	Vazyme	Cat#R223-01
SYBR Green qPCR Master Mix	Vazyme	Cat#Q331-02
ExFect Transfection reagent	Vazyme	Cat#T101-01/02

### Resource availability

#### Lead contact

Further information and requests for resources and reagents should be directed to and will be fulfilled by the lead contact, Zhiqiang Pan (zhiqiangp2002@aliyun.com).

#### Materials availability

All unique reagents generated in this study are available from the lead contact.

#### Data and code availability

• Data reported in this paper can be shared by the lead contact upon request.
• This work does not include any original code.
• Any additional information required to reanalyze the data reported in this paper is available from the lead contact upon request.

### Experimental model and study participant details

#### Mice

Male BALB/c mice (6–8 weeks old) and pregnant female C57BL/6J mice (E12-E13 days) were obtained from the Animal Science Center of Xuzhou Medical University. All procedures performed were approved by the Animal Care and Use Committee of Xuzhou Medical University. Mice were housed in rooms with a controlled environment (temperature: 25 ± 1°C, humidity: 50%–60%, and 12:12 h light: dark cycle). Food and water were available *ad libitum* and the mice assigned to treatment groups randomly.

#### Animal models

The chronic constriction injury (CCI) model, a preclinical neuropathic pain model, was established as described in our previous work.^48^ Briefly, mice were anesthetized with sevoflurane and positioned on a heat mat to avoid hypothermia. The left common sciatic nerve was exposed and three loose ligatures of 4-0 silk thread were placed around the sciatic nerve at intervals of about 1 mm proximal to the trifurcation of the nerve. The muscle and skin incisions were then closed in layers. Finally, the mice were monitored until recovery from anesthesia before being returned to their home cages.

The spared-nerve injury (SNI) model of neuropathic pain was carried out as published previously.^49^^,^^50^ In brief, mice were anesthetized with sevoflurane, and an incision was made on the left hindlimb thigh to expose the three branches of the sciatic nerve. The peroneal and tibia branches were isolated and ligated with 5-0 silk thread. The ligated nerves were then transected and the intact sural nerve was preserved. The incisions in the skin and muscles were closed with 5-0 and 6-0 silk sutures, respectively.

For sham animals, surgery followed the procedures described above for SNI or CCI mice, but without the ligature/transection of the respective nerves.

For the complete Freund’s adjuvant (CFA)-induced model of chronic inflammatory pain, according to previous studies,^51^^,^^52^^,^^53^ a 40-μL solution of CFA (F5881; Sigma-Aldrich, USA) was injected subcutaneously into the plantar surface of the unilateral hind paw.

#### Primary neuron culture

The extraction and culture of primary neurons was performed according to a previous study.^54^ C57BL/6J mice (E12–E13 days) were sacrificed then the brains were harvested and transferred to pre-cooled high-glucose DMEM (Dulbecco’s Modified Eagle’s Medium) (D0821, Sigma Aldrich). Next, the meninges were stripped and the hippocampus was separated and rinsed twice in pre-cooled high-glucose DMEM medium. 0.05% trypsin-EDTA (G4001, Servicebio) was added and the mixture gently brushed to form a suspension of single cells. An equal volume of high-glucose DMEM containing 20% FBS (JR Scientific, Woodland, CA) was added, then the suspension was filtered through a 200-μm mesh sieve and centrifuged at 1,000 rpm for 5 min at 4°C. After the supernatant was discarded, cells were resuspended in Neurobasal complete medium (21103049, Gibco), and seeded at a density of 1×10^5^ cells/cm^2^ in a cell culture plate precoated with poly-L-lysine (Sigma-Aldrich). The cells were cultured in an incubator containing 5% CO_2_ and 95% O_2_ at 37°C.

### Method details

#### Behavioral tests

Male mice were used for behavioral tests. Mechanical test was carried out according to our previous work.^48^ Mice were placed individually in a plexiglas box on an elevated mesh screen and allowed to adapt for 30 min. Then, two calibrated von Frey filaments (0.07 g and 0.4 g, Stoelting, Wood Dale, IL.) were applied to the hind paw for approximately 1 s to measure the paw withdrawal frequency (PWF). This was repeated 10 times for each filament with 5 min interval. Brisk withdrawal or paw flinching was regarded as a positive response. The percent response frequency was calculated as follows: [response frequency = (number of paw withdrawals/10 trials) × 100%].

For the heat test, paw withdrawal latencies to a thermal stimulus were measured with a Model 336 Analgesia Meter (IITC Inc. Life Science Instruments, Woodland Hills, CA), according to the protocol described previously.^55^ First, mice were placed in a plexiglas chamber on a glass plate. A radiant heat source emitted from the light box was applied to the middle of the plantar surface of the hind paw. A quick withdrawal or lifting of the hind paw was taken as the signal to turn off the light. The time from light on to light off was recorded as the paw withdrawal latency. Five trials were conducted on each side, with a 5 min interval between trials. An automatic 20-s cut off was used to avoid tissue damage.

The conditioned place preference (CPP) was performed in a custom-made two-chamber box (length ×width × height, 40 × 20 × 30 cm^3^). The right chamber had black-and-white vertical stripes on the walls and a smooth floor, the left chamber had black-and-white horizontal stripes on the walls and a mesh floor. The CPP test was performed according to a previous study^29^ with some modifications. After microinjection for 7 days, day 1 was the preconditioning test (pre-test) day in which mice were given free access to the two chambers and the time that the mice spent in each chamber was recorded. Mice were not used if they spent more than 80% or less than 20% of the total time in one chamber on the pre-test day. On the days 2–4, the mice were restricted to one chamber and received saline. Approximately 6 h later, the mice were restricted to the other chamber and received 0.8% lidocaine. On day 5 (test day), the mice were allowed to freely explore the chambers for 15 min and the time spent in each chamber was recorded. On the pre-test and test days, the animal’s movement was video-tracked and analyzed online or offline with EthoVision XT video tracking software. We calculated the relative time spent of *Dhx9-shRNA* to scrambled group in the lidocaine-treated side on test day.

For anxiety-like behavior, after *Dhx9*-shRNA microinjection for 7 days or AAV-hSyn-*Dhx9* microinjection for 14 days, the elevated plus maze (EPM) test and open field test (OFT) were performed as previously described.^56^ For EPM, mice were adapted to the room for 1 h before tests. The EPM (Med Associates, St. Albans, Vermont) consisted of The EPM consisted of two open arms (30 × 6 cm), two closed arms (30 × 6 × 15 cm), and a central area (6 × 6 cm), with the open and closed arms being perpendicular to form a cross. For each test, mouse was placed in the center square and allowed to move freely. The number of entries and time spent in each arm was recorded for 5 min. OFT was performed with an open field (40 × 40 × 40 cm). The Activity Monitor system (MED-associates) was used to record locomotor activity. Individual mice were placed in the center of the open field and activity was measured for 10 min.

#### Reagent microinjection

*Dhx9* siRNA, saRNA, Lenti-shRNA and AAV-hSyn-*Dhx9* were prepared and microinjected into the hippocampus according to previously published protocols.^57^^,^^58^ Briefly, mice were anesthetized with sevoflurane and placed in a stereotaxic frame. Under stereotaxic guidance, the animals were microinjected with 1 μL of reagent into the right hippocampus (−2 mm posterior to bregma, 2 mm lateral to the midline, 1.8 mm dorsal from the surface of the skull). In the sham groups, 1 μL of control solution was injected. The glass micropipette was left in place for 10 min after injection to allow virus to fully disperse. Then, the glass micropipette was slowly retracted. The skin wound was closed with 5-0 silk sutures. The sequences for *Dhx9*-siRNA/*Dhx9*-saRNA were synthesized by Genepharma are listed in key resources table. For Lenti-*Dhx9*-shRNA production, synthesized Lenti-*Dhx9*-shF and Lenti-*Dhx9*-shR oligos were annealed and ligated to MluI and ClaI-treated pLVTHM. The constructed plasmids were confirmed by Sanger’s sequencing. According to our previous study,^59^ the constructed core plasmid (8 mg) and two envelope plasmids, PSPAX2 (6 mg) and PMD2G (2 mg), were co-transfected into HEK293T cells in a 10 cm dish. After transfection for 48 h, the supernatant was collected and concentrated by using a Centricon Plus-70 filter unit (UFC910096, Millipore). For AAV-hSyn-Dhx9 production, the AAV-Dhx9 plasmid was obtained from Miao Ling Biotechnology Co. , Ltd. The AAV-Dhx9 plasmid (10 μg) and two envelope plasmids, AAV-DJ (10 μg) and pHelper (10 μg), were co-transfected into HEK293T cells in a 15 cm dish according to manufacturer instructions of ExFect Transfection reagent (T101-01/02, Vazyme). The cells were collected at 72 h after transfection, and concentrated by using AAVpro Purification kit (6666, Takara).

#### Golgi-Cox staining

Golgi staining was conducted according to the manufacturer’s instructions in the PD Rapid GolgiStain Kit (PK401, FD NeuroTechnologies). Images of Golgi-stained neurons were acquired on a Zeiss microscope using z stack images. Only CA1 neurons were included in our analysis. Each neuron was reconstructed using Fiji-Image J (https://imagej.net/Fiji/Downloads), and all analyses were performed using the Simple Neurite Tracer in Plugins in ImageJ; the center of all concentric circles was defined as the center of cell soma. To calculate spine density, a length of basal dendrite was traced and measured, and the spine density was calculated from the number of spines divided by the length of dendrite. The total length of dendritic branches was counted at each order away from the cell body. To assess the complexity of neural dendrites, the number of dendrites that cross a series of concentric circles at 40 μm intervals from the soma was determined by Sholl analysis. Eighteen neurons from 3 animals per condition were analyzed in a blinded manner.

#### Fiber photometry

First, *Dhx9*-shRNA virus was stereotaxically injected into the top of CA1 in mouse hippocampus and then 200 nL of AAV-CaMKIIα-GCaMP6s virus was injected at the same location. A ceramic fiber 200-μm in diameter was implanted at the injection site to transmit excitation light and collect the emitted light. After waiting 2 weeks after injection for viral expression, a recording system was used to measure changes in the population calcium signal from the hippocampus. We set the excitation light wavelength to 470 nm and 410 nm; data from the 410 nm-channel were used as the baseline.

Before fiber photometry recording, mice were allowed to adapt to the cage and surrounding environment for 30 min, then they were connected to the fiber commutator and allowed to acclimatize for another 30 min. We adjusted the channel value to 45 to cover the 200-μm fiber and set the camera parameters as follows: sampling, 30Hz; exposure, 10 ms; gain, 0. To assess the responses of glutamatergic neurons in the hippocampal region to nociceptive von Frey mechanical stimuli, we compared the GCaMP6s signal in the first 3 s of stimulation (F) to the baseline value (F0), and quantified it as [value signal (F)−F0]/F0. We converted GCaMP6s signals to *Z* score trajectories by calculating the mean and standard deviation (SD) of the first 3 s of the GCaMP6s signal then calculating the *Z* score [(F-mean)/SD] of each point in the GCaMP6s signal trajectory. Finally, we recorded 24 trials from 3 animals per condition, and the corresponding heatmap of the *Z* score plot, the area under the curve (AUC), and peak value were analyzed to quantify the response of hippocampal glutamatergic neurons to painful stimuli.

#### Western blotting

Total protein extracts were obtained by lysing the isolated hippocampus in ice-cold lysis buffer (RIPA:cocktail, 100:1). After the protein concentration was measured, the sample was heated for 5 min at 100°C. The level of proteins was detected by SDS/PAGE under reducing conditions. The proteins were electrophoretically transferred onto PVDF membrane. The membrane was blocked with 5% skim milk in washing buffer containing 0.1% Tween 20 for 2 h, then incubated overnight with the following primary antibodies: rabbit anti-DHX9 (1:4,000, 17721-1-AP, Proteintech), mouse anti-β-tubulin (1:5,000, 66240-1-Ig, Proteintech), rabbit anti-PSD95 (1:1,000, R1508-4, HUABIO), rabbit anti-SYP (1:5,000, ab32127, Abcam), or rabbit anti-Histone H3 (1:5,000, ab1791, Abcam). The proteins were detected by HRP-conjugated anti-mouse (1:2,000, A0216, Beyotime) or anti-rabbit secondary antibody (1:2,000, A0208, Beyotime). Then ECL (P0018S, Beyotime) was used to detect the protein band and all blot images were quantified in ImageJ 2.0.0.

#### Immunofluorescence

Mice were anesthetized with sevoflurane, and brains were cut on a sliding freezing stage microtome in 30 μm thick sections. Hippocampal sections were washed 3 times in PBS. Following 15 min permeabilization with 0.3% Triton X-100 in 0.01 M PBS, sections were blocked with 10% donkey serum for 2 h. Next, sections were incubated overnight at 4°C with the following primary antibodies: DHX9 (1:300, 17721-1-AP, Proteintech), NeuN (1:200, ab104224, Abcam), Iba1 (1:200, ab5076, Abcam), GFAP (1:200, ab3554, Abcam), MAP2 (1:200, ab11267, Abcam), FMRP (1:100, sc-101048, Santa Cruz), STAU1 (1:100, sc-390820, Santa Cruz), or c-Fos (1:200, ab208942, Abcam). The next day, slides were washed 3 times for 7 min in PBS. Then, sections were incubated with the corresponding fluorescent-conjugated secondary antibody (A32766, A32744, A32790, A32754, Invitrogen) for 2 h at room temperature in the dark. DAPI Fluoromount-G aqueous mounting medium with anti-fade properties (SouthernBiotech) was used to identify cell nuclei. Slides were stored in the dark at 4°C until needed for analysis. Fluorescence images were captured with an Olympus FV1000 laser confocal microscope. For c-Fos positive neuron analysis, ImageJ was used to analysis the signals of hippocampal neurons from 3 animals per condition.

#### RNA immunoprecipitation (RIP)

Cytosolic extracts from the hippocampus were prepared as described in the manufacturer’s instructions in the Nuclear and Cytoplasmic Extraction Kit (CW0199S, CoWin Biosciences). Cytoplasmic protein was divided into three groups: Input, DHX9, and IgG (negative control). The Input group was temporarily stored in a −80°C freezer and the remaining two groups were subjected to non-contact ultrasonic lysis, "M" strength, 5 min, 3 times. Then 1 μL rabbit IgG and 20 μL Protein A + G Agarose beads were added and the samples were incubated at 4°C for 2 h. After blocking, the supernatant was collected. Next, rabbit anti-DHX9 antibody or normal rabbit IgG antibody (1 μg, A7016, Beyotime) was added to the corresponding samples, which were then rotated at 4°C overnight. Next, protein A + G agarose gels (P2055, Beyotime) were added and the samples were incubated with rotation for 1 h at 4°C. The samples were washed three times with RIP wash buffer. Finally, RNA was eluted with TE buffer and extracted with Trizol (R401-01, Vazyme). Reverse transcription was followed by qPCR, and *U6* RNAs was used as target reference control.

#### Quantitative polymerase chain reaction (qPCR)

qPCR was performed in QuantStudio 7 Flex. Total RNA was extracted with Trizol (15596026, Invitrogen). mRNAs were reverse transcribed using a HiScript II Q RT SuperMix for qPCR (+gDNA wiper) (R223-01, Vazyme) and quantified using SYBR Green qPCR Master Mix (Q331-02, Vazyme). U6 was used as an internal control. The primers used to amplify mRNA transcripts were synthesized by Sangon Biotech and listed in key resources table.

#### Co-immunoprecipitation (Co-IP)

Co-IP assay was performed using Catch & Release v2.0 Reversible Immunoprecipitation System (17–500, Millipore) according to the manufacturer’s instructions. The antibody DHX9, STAU1 and FRMP were used in co-IP examination, and murine IgG or rabbit IgG was used as negative control.

#### Enzyme linked immunosorbent assay (ELISA)

The level of DDN in hippocampus extracts was analyzed using commercially available Mouse DDN ELISA kit (MM-47575M2, Jiangsu Meimian Industrial Co., Ltd), according to manufacturer’s instructions.

### Quantification and statistical analysis

All data are shown as mean ± S.E.M. Significance was assessed using Student’s *t* tests for comparison of two groups. One-way ANOVAs followed by the Holm–Sidak test were used for comparison of three or more groups. Behavioral data collected at multiple, sequential time points were analyzed using two-way repeated-measures ANOVAs, followed by the Holm–Sidak post hoc test. Statistical analyses were performed in Prism (GraphPad 8.0). Statistical significance was set at p < 0.05.
